# Correlation of antiangiogenic, antioxidant and cytotoxic activities of some Sudanese medicinal plants with phenolic and flavonoid contents

**DOI:** 10.1186/1472-6882-14-406

**Published:** 2014-10-20

**Authors:** Loiy Elsir A Hassan, Mohamed B Khadeer Ahamed, Aman S Abdul Majid, Hussein M Baharetha, Nahdzatul S Muslim, Zeyad D Nassar, Amin MS Abdul Majid

**Affiliations:** EMAN Research and Testing Laboratory, School of Pharmaceutical Sciences, Universiti Sains Malaysia, Kragujevac, Penang Malaysia; Department of Botany, Faculty of Science& Technology, Omdurman Islamic University, P.O. Box 383, Omdurman, Sudan; Advanced Medical and Dental Institute (IPPT), Universiti Sains Malaysia, Kragujevac, Penang Malaysia; Department of Pharmacy, College of medicine and Health Sciences, Hadhramout University, Fuluk, Mukalla, Hadhramout, Republic of Yemen; School of Pharmacy, The University of Queensland, 20 Cornwall Street, Woolloongabba, QLD 4102 Australia

**Keywords:** Antiangiogenesis, Raorta ring assay, Antioxidants, Anticancer, Sudanese medicinal plants, Traditional medicine

## Abstract

**Background:**

Consumption of medicinal plants to overcome diseases is traditionally belongs to the characteristics of most cultures on this earth. Sudan has been a host and cradle to various ancient civilizations and developed a vast knowledge on traditional medicinal plants. The present study was undertaken to evaluate the antioxidant, antiangiogenic and cytotoxic activities of six Sudanese medicinal plants which have been traditionally used to treat neoplasia. Further the biological activities were correlated with phytochemical contents of the plant extracts.

**Methods:**

Different parts of the plants were subjected to sequential extraction method. Cytotoxicity of the extracts was determined by dimethylthiazol-2-yl)- 2,5diphenyl tetrazolium bromide (MTT) assay on 2 human cancer (colon and breast) and normal (endothelial and colon fibroblast) cells. Anti-angiogenic potential was tested using *ex vivo* rat aortic ring assay. DPPH (1,1-diphenyl-2-picrylhydrazyl) assay was conducted to screen the antioxidant capabilities of the extracts. Finally, total phenolic and flavonoid contents were estimated in the extracts using colorimetric assays.

**Results:**

The results indicated that out of 6 plants tested, 4 plants (*Nicotiana glauca*, *Tephrosia apollinea*, *Combretum hartmannianum* and *Tamarix nilotica*) exhibited remarkable anti-angiogenic activity by inhibiting the sprouting of microvessels more than 60%. However, the most potent antiangiogenic effect was recorded by ethanol extract of *T. apollinea* (94.62%). In addition, the plants exhibited significant antiproliferative effects against human breast (MCF-7) and colon (HCT 116) cancer cells while being non-cytotoxic to the tested normal cells. The IC_50_ values determined for *C. hartmannianum*, *N. gluaca* and *T. apollinea* against MCF-7 cells were 8.48, 10.78 and 29.36 μg/ml, respectively. Whereas, the IC_50_ values estimated for *N. gluaca, T. apollinea* and *C. hartmannianum* against HCT 116 cells were 5.4, 20.2 and 27.2 μg/ml, respectively. These results were more or less equal to the standard reference drugs, tamoxifen (IC_50_ = 6.67 μg/ml) and 5-fluorouracil (IC_50_ = 3.9 μg/ml) tested against MCF-7 and HCT 116, respectively. Extracts of *C. hartmannianum* bark and *N. glauca* leaves demonstrated potent antioxidant effect with IC_50s_ range from 9.4–22.4 and 13.4–30 μg/ml, respectively. Extracts of *N. glauca* leaves and *T apollinea* aerial parts demonstrated high amount of flavonoids range from 57.6–88.1 and 10.7–78 mg quercetin equivalent/g, respectively.

**Conclusions:**

These results are in good agreement with the ethnobotanical uses of the plants (*N. glauca*, *T. apollinea*, *C. hartmannianum* and *T. nilotica*) to cure the oxidative stress and paraneoplastic symptoms caused by the cancer. These findings endorse further investigations on these plants to determine the active principles and their mode of action.

## Background

Cancer is a major public health burden in both developed and developing countries. The International Agency for Research on Cancer (IARC), the specialized cancer agency of the World Health Organization reported that about 14.9 million cancer cases were estimated around the world in 2013, of these 7.7 million cases were in men and 6.9 million in women and further this number is expected to increase to 24 million by 2035 [1]. Treating cancer has become a major challenge as there is no single effective treatment that works for all types of cancer. Most of conventional chemotherapy regimens which employ different combinations of cytotoxic drugs which are often associated with serious side effects and chemoresistance. Conventional therapy has also become less favorable in the mindset of sufferers and as a result many patients resort to seeking alternative treatments [2]. The resistance of metastatic cancerous cells to chemotherapy and its adverse effects has become a serious challenge in cancer research. Despite the intensive progress in chemotherapeutics in the last decades, the need to discover and to develop new, alternative, or adjuvant therapeutic agents remains.

Botanicals have long been used traditionally in treatment of various types of cancers [3] and often less associated with the side effects like the modern chemotherapy has [4]. Realizing the potential benefits of botanicals as a source of active anticancer compounds, the National Cancer Institute (U.S.A) collected about 35,000 plant samples from 20 countries and has screened around 114,000 extracts for anticancer activity [5]. Out of the 92 anticancer drugs marketed prior to 1983 in US and among the ones sold worldwide between 1983 and 1994, 60% are of natural origin [6]. This includes natural products, derivatives of natural products or semi-synthetic pharmaceuticals based on natural products models [7].

Angiogenesis is the sprouting of new blood vessels from pre-existing vessels and is strongly implicated in solid tumorgenesis, proliferative retinopathies, obesity and rheumatoid arthritis [8]. Tumor angiogenesis is the consequence of an angiogenic imbalance in which proangiogenic factors predominate over antiangiogenic factors. Furthermore, angiogenesis is essential for growth and metastasis of malignant tumors. Vascular Endothelial Growth Factor-A (VEGF-A) is believed to be a critical angiogenic mitogen [9]. Therefore, tumor angiogenesis can be considered as an important pharmacological target for cancer prevention and treatment [10, 11]. Consequently, this hypothesis has paved a pathway for the development of the cutting edge therapeutic technology called angiotherapy. Anti-angiogenic approach can overcome the cytotoxic adverse effects and chemoresistant problems associated with the classical chemotherapies. Anti-angiognenic drugs work by inhibiting the synthesis of new blood vessels that supply blood, nutrients and oxygen to growing tumor. Previous reports of Avastin, a monoclonal antibody for VEGF, and fluorouracil-based combination therapy showed a significant improvement in survival of patients with metastatic colorectal carcinoma [12]. However conventional antiangiogenic compounds based on monoclonal antibody technology may have limitations from the cost point of view. Plant sources of antiangiogenic compounds have been explored as they are more economical to produce in large scale [13]. However at present there are no plant based antiangiogenic compounds available commercially in the market.

Plants have many phytochemicals which are potential source of natural antioxidants, such as phenolic diterpenes, flavonoids, tannins and polyphenolic acids [14] with versatile biological activities. Plant polyphenolics have been recognized as the potential therapeutic agents targeting cancer, pathological angiogenesis and cardiovascular disease in the next decade [15, 16]. These benefits have been attributed to the presence of antioxidant-rich polyphenolic compounds [16, 17].

The use of traditional medicine especially medicinal plants in Sudan is still the main alternative therapy which is based entirely on the indigenous knowledge gained from ancestral experience. Although, there is an important local ethno botanical biography describing the most frequently used plants in treatment of various clinical conditions however, very few have been studied scientifically for chemometric analysis of the medicinal herbs to identify the active principles. The vast majority is still unexplored phytochemically and their medicinal properties have not yet validated [18].

Compelling data implicate angiogenesis and tumor-associated neovascularization as a central pathogenic step in the process of tumor growth, invasion and metastasis. Subsequently, it was shown that a significant correlation existed between the degree of tumor angiogenesis (micro vessel density) and survival in patients presenting with lymph node-negative breast carcinoma [19]. Therefore, it comes to handy that targeting tumor angiogenesis using antiangiogenic agents the blood vessels which supply the tumor with nutrients and oxygen, in turn it could lead to halt tumor growth and metastasis.

With this background, the present study was undertaken to analyze the antiangiogenic, antioxidant and cytotoxic properties of 32 extracts prepared from six Sudanese plants; *Indigofera spinosa* Forsk. (Leguminosae), *Nicotiana glauca* var. (Solanaceae), *Tephrosia apollinea* (Del.) Link (Leguminosae), *Tamarix nilotica* (Ehrenb.) Bunge (Tamaricaceae), *Combretum hartmannianum* Schweinf. (Combretaceae) and *Capparis decidua* (Forsk.) Edgew (Capparaceae). This study is the first to report the antiangiogenic properties of these selected Sudanese medicinal plants and correlated the activity with antioxidant property. In addition, an investigation on the cytotoxicity of the extracts was conducted to identify the potential source of antineoplastic agents.

## Methods

### Plant material

Six Sudanese medicinal plants, *I. spinosa, N. glauca, T. apollinea, T. nilotica, C. hartmannianum* and *C. decidua* were selected for the study. Plant material was collected during the period of March-July 2013 except *C. hartmaniunum* which was collected during March 2014 from Elgadarif City – Sudan. The taxonomic authentication of all the plants was carried out at The Medicinal and Aromatic Plants Research Institute, National Center for Research by Dr. Wail Alsadig. Voucher specimens (voucher references numbers: MAPRI/NB-53a-g) were deposited at the herbarium of the institute.

### Preparation of extracts

The plant materials were dried in oven (35–40°C) and powdered mechanically. The pulverized plant material (50 g) was subjected to sequential extraction method started with n-hexane and followed by ethanol, methanol and water. All the extracts were prepared by 250 ml of the solvents using hot maceration (40°C) method with intermittent shaking. The extracts were filtered and concentrated at 45°C under vacuum by rotary evaporator (Buchi, USA) and further dried overnight at 45°C. Stock solutions of the extracts were prepared at 10 mg/ml in 100% dimethyl sulfoxide (DMSO). Further serial dilution of the stock was performed with cell culture media to obtain a range of desired concentrations of the extracts. All solvents used in this study were of analytical grade.

### Experimental animals

Twelve to fourteen weeks old healthy Sprague Dawley male rats were used. To avoid physiological variations that could affect the process of angiogenesis in female rats due to estrous cycle, only male rats were used in rat aortic ring assay. The animals obtained from animal house facility of Universiti Sains Malaysia (USM) and were kept for one week in animal transit house (School of Pharmaceutical Sciences, USM) prior to the experiments. The animals were kept in well ventilated cage with food and water provided. The animals were euthanized using CO_2_ and dissected to excise thoracic aorta. All procedures were carried out according to the guidelines of Animal Ethics Committee USM. The present study was submitted to the institutional animal ethics committee, “Animal Ethics Committee USM” for evaluation and the present study is approved by the committee (approval Reference number: PPSG/07 (A)/044/(2010) (61)).

### Chemicals and reagents

Cell culture reagents were purchased from Gibco, USA; RPMI 1640 medium; catalogue number (A10491-01), Dulbecco’s Modified Eagle Medium; Catalogue number (31100–035) were obtained from GIBCO, UK. Phosphate buffered saline, trypsin, heat inactivated foetal bovine serum (HIFBS), penicillin/streptomycin (PS), fibrinogen, aprotinin, thrombin, suramin, aprotinin, 6-Aminocaproic acid, L-glutamine, thrombin and gentamicin were purchased from Sigma, Germany. MTT (3-(4,5-Dimethylthiazol-2-yl)- 2,5diphenyl tetrazolium bromide) was procured from Sigma-Aldrich, USA. Dimethyl sulfoxide (DMSO) was purchased from Fluka, USA.

### Cell lines and culture conditions

Human Umbilical Vein Endothelial Cell line HUVEC (Passage No. 3), catalogue number (C2517A); human colorectal carcinoma cell line HCT-116 (Passage No. 5), catalogue number (CCL-247); human hormone sensitive and invasive breast cancer cell line MCF-7 (Passage No. 4), catalogue number (HTB-22); human colorectal normal cell line CCD-18 (Passage No. 3), catalogue (CRL-1459) were purchased from ScienCell, USA. HUVEC were maintained in endothelial cell medium (ECM) (ScienCell, USA) supplemented with endothelial cell growth supplements (ECGS), 5% HIFBS and 1% PS. HCT-116 cells were maintained in RPMI whereas, MCF-7 and CCD-18Co were maintained in DMEM medium. The media were supplemented with 5% heat inactivated fetal bovine serum and 1% penicillin/streptomycin. Cells were cultured in a humidified incubator at 37°C supplied by 5% CO_2_. Cell culture work was done in sterile conditions using Class II biosafety cabinet (ESCO, USA).

### Rat aorta ring assay

This assay was carried out on rat aortic explants as previously described [20]. Thoracic aortas were removed from euthanized male rats, rinsed with serum free medium and cleaned from fibroadipose tissues. Totally 18 rats were used in this assay and approximately 12 to 14 rings (each ring is about 1 mm thickness) were prepared from an each aorta. The aortas were cross sectioned into small rings and seeded individually in 48-wells plate in 300 μL serum free M199 media containing 3 mg/ml fibrinogen and 5 mg/ml aprotinin. Ten microliters of thrombin (50 NIH U/ml in 1% bovine serum albumin in 0.15 M NaCl) was added into each well and incubated at 37°C for 90 min to solidify. A second layer (M 199 medium supplemented with 20% HIFBS, 0.1% έ-aminocaproic acid, 1% L-Glutamine, 2.5 μg/ml amphotericin B, and 60 μg/ml gentamicin) was added into each well (300 μL/well). All the extracts were added at final concentrations of 100 μg/ml. Suramin and 1% DMSO were used as positive and negative controls, respectively. On day four, the medium was replaced with a fresh one containing the test materials. On day five, aortic rings were photographed using EVOS f1 digital microscope (Advanced Microscopy Group, USA) (40× magnification) and subsequently the length of blood vessels outgrowth from the primary tissue explants was measured using Leica Quin software.

The inhibition of blood vessels formation was calculated using the formula;

% blood vessels inhibition = [1- (A0/A)] × 100, Where; A0 = distance of blood vessels growth in treated rings in μm, A = distance of blood vessels growth in the control in μm.

The results are presented as mean percent inhibition ± SEM, (*n* =8).

The significant difference between the microvessels out growth in treated versus untreated aortic rings was calculated using Student’s t test. Based on the results of this assay, TAF273 was chosen for the subsequent investigations for the anti-angiogenic property.

### Cytotoxicity assay

The MTT cytotoxicity assay was performed according to the method previously described [21]. Cells were seeded at 1.5 × 10^4^ cells in each well of 96-well plate in 100 μl of fresh culture medium and were allowed to attach for overnight. For screening, the cells (70 - 80% confluency) were treated with the extracts at the final concentration of 50 μg/ml. Later on, in order to obtain a dose–response curve, the most active extracts were tested for cytotoxicity at 3.12, 6.25, 12.5, 25, 50 and 100 μg/ml concentrations. After 48 h of the treatment the medium was aspirated and the cells were exposed to MTT solution prepared at 5 mg/ml in sterile PBS was added to each well at 10% v/v in the respective medium and was incubated at 37°C in 5% CO_2_ for 3 h. The water insoluble formazan salt was solubilized with 200 μl DSMO/well. Absorbance was measured by infinite® Pro200 TECAN Group Ltd., (Switzerland) at primary wave length of 570 nm and reference wavelength of 620 nm. Each plate contained the samples, negative control and blank. DMSO (1% v/v) was used as a negative control. 5-fluorouracil, Tamoxifen and Betulinic acid were used as standard reference control for HCT 116, MCF-7 and CCD-18Co cell lines, respectively. The assay was performed in quadricate and the results were presented as a mean percent inhibition to the negative control ± SEM.

### Determination of total phenols

Total phenols in the extracts were determined by a colorimetric method as described by Al-Suede and co-workers [22]. A stock of 1 mg/ml of extracts was prepared in methanol and 100 μl of each extract was added separately to 750 μl of Folin-Ciocalteau phenol reagent (1:10 diluted with double distilled H_2_O). After 5 min incubation in the dark at room temperature, 750 μl sodium bicarbonate solution (60 g/l) was added and incubated at 30°C in the dark for 90 min. The absorbance was measured at 725 nm using TECAN Multi-mode microplate reader Model Infinite® 200 (Mannedorf, Switzerland). Gallic acid was used (5–80 μg/ml) to construct the standard calibration curve. The results were expressed as Gallic acid equivalents per 100 mg of extract (mg GAE/100 mg).

### Determination of total flavonoids

The total flavonoids content in the extracts was determined using aluminum chloride colorimetric method with quercetin as standard [23]. A solution of 4 mg/ml of quercetin in methanol was prepared. Exactly, 500 μl of different concentrations (3 to 200 μg/ml) of the extracts were taken in separate test tubes. To each of the test tubes, 0.1 ml of 10% (w/v) aluminum chloride solution, 0.1 ml of 1 M potassium acetate solution, 1.5 ml of methanol and 2.8 ml of distilled water was added. The test tubes were thoroughly mixed and after incubation at room temperature for 30 min, the absorbance reading of the reaction mixture was measured at 415 nm using a spectrophotometer (Perkin Lambda 45). A standard curve plotting all the different concentrations of quercetin standard was constructed and the total flavonoid content is expressed as micrograms of quercetin equivalent. The data were presented as mean ± SEM (n =6).

### DPPH scavenging effect

DPPH (1,1-diphenyl-2-picrylhydrazyl) assay was carried out to evaluate the scavenging activity of the extracts [24]. The stock solution of DPPH was prepared at a concentration of 200 μM in absolute methanol while stock solutions of the extracts were prepared at concentration of 10 mg/ml. DPPH was dispensed into 96-well plate (100 μl/well) and immediately, 100 μl of test samples were added at final concentrations of 12.5, 25, 50, 100, 200 μg/ml. Methanol alone and methanol with DPPH were used as blank and negative control, respectively. Ascorbic acid was used as positive control. The mixtures were incubated at 30°C for 30 min in the dark and then the absorbance was measured at 517 nm using TECAN microplate reader Model Infinite® 200 (Mannedorf, Switzerland). The dose response curves were obtained and then used to calculate the median inhibitory concentration (IC_50_). The results are expressed as mean ± SEM (n =6).

## Results

### Plant extraction

Four exacts were prepared from each plant material, starting with n-hexane followed by ethanol, methanol and water. The yield of each extract was calculated and presented in Table 1 as w/w percent yield. Among all the extracts, hexane extracts of all the tested plants produced the lowest yield except for *N. gluaca* leaves extract (7.8%). On average, the ethanol extracts of the tested plants showed the highest yield followed by methanol and water extracts. For instance, the highest yield recorded was 15.68% for ethanol extract of *C. hartmannianum* leaves. Table 1 shows the list of plants and the parts used in this study.

**Table 1:** **Parameters of different extracts of the selected Sudanese plants**

Botanical name	Local name	Used part	Solvent	Texture	Yield %
*Indgosfera spinosa*	Hipatoheep	Stem	n-Hexane	Sticky	0.8
			Ethanol	Solid	2.82
			Menthol	Solid	1.72
			Water	Powder	3.36
*Nicotiana gluaca*	Hili leasa	Leaves	n-Hexane	Sticky	7.8
			Ethanol	Gummy	11.73
			Menthol	Gummy	4.11
			Water	Powder	1.698
		Stem	n-Hexane	Gummy	0.82
			Ethanol	Gummy	6.21
			menthol	Gummy	3.94
			water	Powder	5.94
*Tephrosia apollinea*	Dhawasi	Aerial parts	n-Hexane	Gummy	0.6
			Ethanol	Sticky	2.41
			Menthol	Sticky	5.86
			Water	Powder	4.01
*Tamarix nilotica*	Tarffa	Leaves	n-Hexane	Gummy	1.4
			Ethanol	Gummy	12.64
			Menthol	Gummy	4.14
			Water	Powder	7.41
*Combretum hartmannianum*	El-Habeel	Leaves	n-Hexane	Gummy	7.16
			Ethanol	Sticky	15.68
			Menthol	Gummy	6.64
			Water	Powder	6.04
		Bark	n-Hexane	Gummy	0.163
			Ethanol	Solid	9.18
			Menthol	Solid	3.37
			Water	Powder	2.66
*Capparis decidua*	Tunduli	Stem	n-Hexane	Gummy	0.892
			Ethanol	Gummy	6.81
			Menthol		3.37
			Water	Powder	01.56

### Inhibitory effect of the extracts on sprouting of microvessels in rat aortic explants

**Figure 1:**
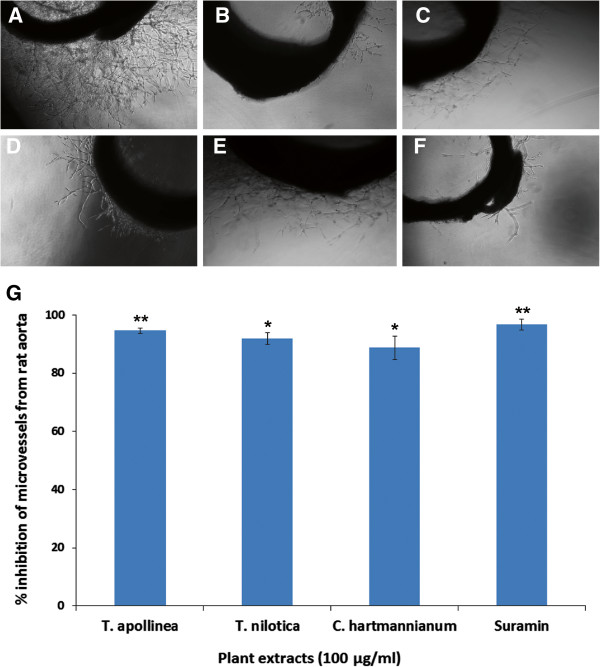
**Antiangiogenic effect of selected Sudanese plants (100 μg/ml) against sprouting of microvessels in rat aortic explants. A)** Photomicrographic image of rat aortic ring of negative control showing extensive growth of microvessels. **B)** Photomicrographic image of rat aortic ring representative of polar extract of *T. apollinea* displaying potent inhibition of growth of microvessels. **C)** Treatment with *T. nilotica* showed significant inhibitory effect against sprouting of microvessels from rat aortic rings. **D)** Photomicrographic image of rat aortic explant treated with *C. hartmannianum* extract demonstrated considerable effect of antiangiogenicity. **E)** Photomicrographic image of rat aortic explant treated with non-polar extract of *T. apollinea* showing significant inhibitory effect on growth of microvessels. **F)** Photomicrographic image of rat aortic explant treated with standard reference, suramin exhibiting strong inhibitory effect on microvessels growth. **G)** Graphical representation of antiangiogenic activity of the active extracts obtained from the selected Sudanese plants. The values are expressed as mean ± SEM (n =10). **p < 0.01 and *p < 0.05 compared to negative control group (0.1% DMSO).

This assay was performed as the primary assay to screen the antiangiogenic potential of the extracts. Figure 1A shows a massive sprouting of microvessels in the aortic explants of vehicle treated group (negative control). Plant extracts with more than 60% inhibition of sprouting of blood vessels were considered as active extracts. Table 2 depicts the antiangiogenic properties of plant extracts determined by rat aorta ring assay. Out of 32 extracts, 12 extracts from three plants namely *T. apollinea*, *C. hartmannianum* and *T. nilotica* showed potent (more than 60%) inhibitory activity. The highest inhibition (100%) was produced by ethanol extract of aerial parts of *T. apollinea* (Figure 1B), followed by water extracts of *T. nilotica* (Figure 1C). Interestingly, all the extracts of *C. hartmannianum* (Figure 1D) stem bark displayed significant (*p <0.05*) inhibition of micro-blood vessels with an order of ethanol (88.74%) followed by menthol (86.68%) and n-hexane (81.75%). Moreover, methanol and ethanol extracts of *C. hartmannianum* leaves also demonstrated significant (*p <0.01* and *p <0.05*, respectively) antiangiogenic effect with 68.84 and 63.68%, respectively. In addition, Figure 1E depicted the remarkable inhibitory effect on microvessel growth from the rat aortic explant treated with *T. nilotica* extract. These results were very much comparable with the positive control, suramin which demonstrated potent inhibition of microvessel growth (Figure 1F). Figure 1G graphically depicts the difference between the effects of the extracts and Suramin.

**Table 2:** **Antiangiogenic activity of the selected Sudanese plants on rat aortic explants**

Botanic name ^n^	Part used	Extract	Inhibition (%)
*Tephrosia apollinea*	Aerial parts	Ethanol	94.6 ± 1.8**
*Tamarix nilotica*	Leaves	Water	91.7 ± 2.2**
*Combretum hartmannianum*	Bark	Ethanol	88.7 ± 1.6*
*Combretum hartmannianum*	Bark	Methanol	86.6 ± 3.8**
*Combretum hartmannianum*	Leaves	n-hexane	81.7 ± 1.7*
*Tephrosia apollinea*	Aerial part	Methanol	79.8 ± 2.6*
*Tephrosia apollinea*	Aerial parts	n-hexane	73.1 ± 1.5*
*Combretum hartmannianum*	Bark	Water	71.5 ± 5.3*
*Tamarix nilotica*	Leaves	Methanol	65.5 ± 2.9*
*Combretum hartmannianum*	Leaves	Methanol	68.8 ± 2.2*
*Combretum hartmannianum*	Leaves	Ethanol	63.6 ± 1.4*

Results are presented as mean percent inhibition ± S.D, n = 3.

Statistical significance is expressed as * = *P* <0.05, ** = *P <*0.01.

### Anti-proliferative effect of the extracts against cancer cells

The MTT assay was used to screen the possible cytotoxic activity of 32 extracts against two human cancer cells lines (HCT-116 and MCF-7) and two normal cell lines (HUVEC and CCD-18Co). For screening, the cells were treated with the extracts at 50 μg/ml concentration. The extracts with more than 60% inhibition of cell proliferation were considered as active extracts. Hexane extracts of *N. glauca* leaves and stem exhibited the highest cytotoxicity on all the tested cell lines, while hexane and ethanol extracts of aerial part of *T. apollinea* showed selective antiproliferative effect against breast cancer cell line (MCF-7) with 87.20 and 86.94%, respectively. The hexane extract of *C. hartmannianum* leaves potently inhibited the growth of both cancer cell lines (HCT-116 and MCF-7) with 80.03 and 95.76% anti-proliferative effect, respectively. Moreover, the ethanol extract of *C. hartmannianum* stem bark showed selective cytotoxicity towards MCF-7 with 76.79%. Interestingly, all the extracts of *C. hartmannianum* showed poor cytotoxicity against the normal cell lines (Table 3). Further, the most active extracts were selected to study the dose response cytotoxic effect. The median inhibitory concentration (IC_50_) values for the most active extracts and the respective standard reference drugs were calculated for all the tested cell lines and the values are given in Table 4.

**Figure 2:**
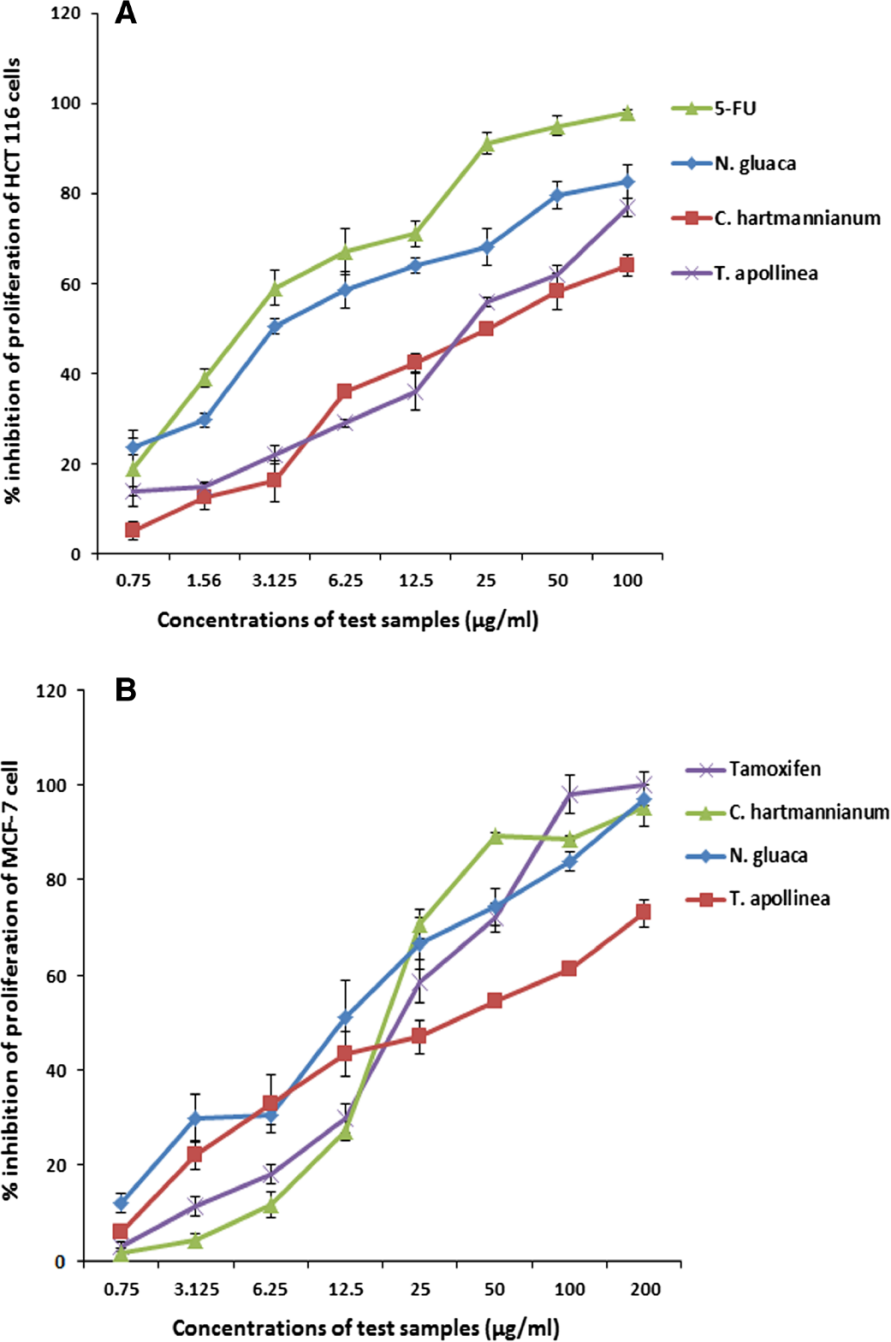
**Dose dependent inhibitory effect of the active extracts of the selected Sudanese plants against HCT 116 (A) and MCF-7 (B) cell lines.** The values are expressed as mean ± SEM (n =6).

The results were comparable with the respective standard reference drugs, 5-fluorouracil, betulinic acid and tamoxifen. Figures 2A and B show the graphical illustration of the dose-dependent antiproliferative effect of the active extracts against human HCT 116 and MCF-7 cell lines.

**Figure 3:**
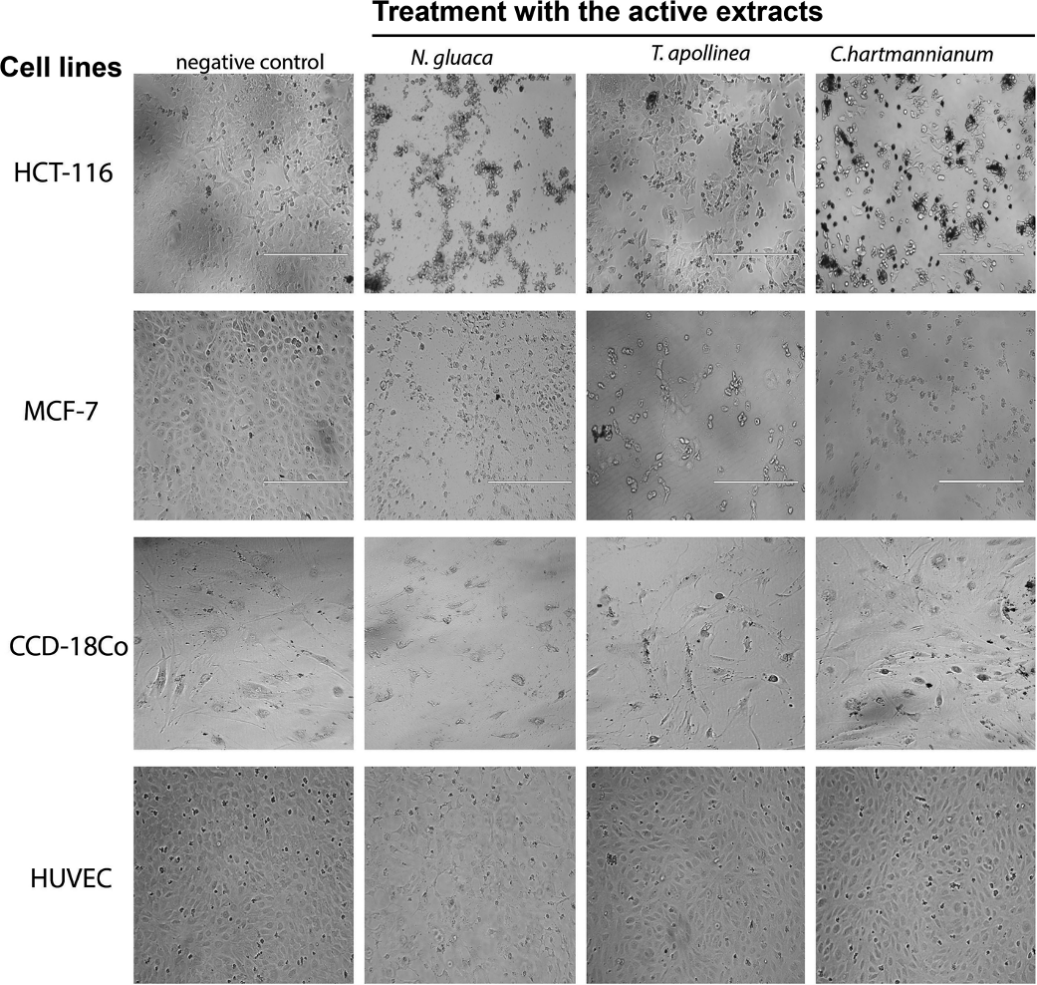
**Photomicrographic images of the cancer (HCT 116 and MCF-7) and normal (HUVEC and CCD-18Co) cell lines taken under an inverted phase-contrast microscope at × 200 magnification with a digital camera at 48 hours after treatment with the active extracts of the selected Sudanese plants.** The controlled cells showed a fully confluent growth with the compact layer of proliferating cells. Whereas, treatment with the active extracts caused a drastic reduction in the density of cell-population however, treatment with the extracts did not produced significant cytotoxicity against the tested normal cells (HUVEC and CCD-18Co) when compared to the negative control group. The picture revealed that the cells revealed sever morphological changes in their native cellular characteristics. Treatment caused the cells to lose the psuedopodial like membrane projections. Higher magnification of the photomicrographs revealed several clear features of apoptosis, such as the membrane blebbing, nuclear condensation and apoptotic bodies in the treated cells.

Figure 3 shows the photomicrographic images of the treated HCT 116, MCF-7, CCD-18Co and HUVEC cell lines. The morphological feature of the treated cancer cells presented clear evidence of strong cytotoxicity of the extracts, as the vehicle (1% DMSO) treated cells displayed a compact monolayer of aggressively growing cancer cells with prominent nuclei and intact cell membrane. Whereas the images taken from the extracts treated group showed a drastic reduction in the number of cells because of the anti-proliferative activity of the extracts. In addition, the extracts severely affected the pseudopodial projections of the cells which rendered the cells non-adherent and become round shaped. Interestingly, all the extracts studied showed either mild or negligible cytotoxicity towards the both normal (HUVEC and CCD-18Co) cell lines which were used as the model cell lines for the normal human cells.

**Table 3:** **Cytotoxic effect of different extracts of the selected Sudanese plants**

Plants	Part used	Solvent used	% inhibition of cell proliferation			
			HCT-116	MCF-7	CCD	HUVECs
*Indgosfera spinosa*	Stem	n-Hexane	27.79	40.43	4.16	5.64
		Ethanol	14.98	42.48	5.42	6.08
		Methanol	11.16	41.86	4.93	6.79
		Water	12.95	33.90	6.18	8.11
*Nicotiana gluaca*	Leaves	n-Hexane	95.08	99.38	45.63	62.05
		Ethanol	49.66	81.10	38.57	36.25
		Methanol	19.83	75.58	10.15	22.64
		Water	17.20	49.81	8.09	19.79
	Stem	n-Hexane	87.07	92.01	44.35	59.46
		Ethanol	33.58	49.28	32.63	25.43
		Methanol	43.47	53.51	40.47	27.87
		Water	28.95	57.53	38.56	19.89
*Tephrosia apollinea*	Aerial part	n-Hexane	53.86	87.20	9.65	26.95
		Ethanol	46.91	86.94	7.88	16.28
		Methanol	47.43	87.09	10.23	18.91
		Water	16.86	25.60	4.68	5.79
*Tamarix nilotica*	Leaves	n-Hexane	29.33	47.25	8.77	14.58
		Ethanol	7.8	46.61	5.83	22.89
		Methanol	19.61	46.14	6.65	17.35
		Water	6.93	28.47	3.87	11.68
*Combretum hartmannianum*	Leaves	n-Hexane	80.03	95.76	7.75	16.62
		Ethanol	33.69	54.04	6.35	7.88
		Methanol	17.61	78.22	7.79	9.58
		Water	26.02	36.30	6.23	5.65
	Bark	n-Hexane	26.30	42.43	5.74	6.61
		Ethanol	29.11	76.79	6.48	8.36
		Methanol	13.75	73.64	9.57	11.59
		Water	28.40	49.92	10.11	9.47
*Capparis decidua*	Stem	n-Hexane	33.78	50.07	5.34	15.45
		Ethanol	8.5	41.99	3.69	5.77
		Methanol	14.76	39.87	5.58	19.57
		Water	21.82	54.41	6.76	4.38

**Table 4:** **IC**_**50**_ **(μg/ml) values of the active extracts of the selected Sudanese plants**

Extracts	Carcinoma cell lines		Normal cell lines	
	HCT 116	MCF-7	HUVEC	CCD-18CO
*C. hartmannianum*	27.2	8.48	116.5	466.4
*N. gluaca*	5.4	10.78	46.2	79.82
*T. apollinea*	20.2	29.36	98.4	345.6
Positive controls	5-Flourouracil	Tamoxifen	Betulinic acid	Betulinic acid
	3.9	6.67	88.4	128.9

### Total phenolic contents in the extracts

The amount of total phenolic compounds present in each extract was determined from linear regression equation of calibration curve, {y =0.0034 + 0.0144 (R^2^ = 0.9976) and expressed as Gallic acid equivalent in mg/ml of extracts. Table 5 depicts the result of assessment of total phenolic contents in all the tested extracts. It is found that, hexane extracts of all the plants were deprived of total phenolic contents, either no or negligible amount of phenolic contents were detected in all hexane extracts. However, the methanol, ethanol and water extracts of the tested plants showed considerable level of phenolic contents. The results showed that, both stem bark and leaves of *C. hartmannianum* displayed high contents of total phenolics in which ethanol, methanol and water extracts showed 404.05 ± 0.06, 96.56 ± 0.05 and 523.36 ± 0.00 mg GAE/g, respectively for stem bark, whereas 169.19 ± 0.02, 392.83 ± 0.01, 268.21 ± 0.03 mg GAE/g, respectively for leaves. The results showed that methanol and water extracts of *T. nilotica* also demonstrated significant (*p <0.05*) level of phenolic contents with 333.96 ± 0.01 and 171.66 ± 0.02 mg GAE/g, respectively. The leaves extracts of *N. glauca* showed moderate level of total phenolic contents (ethanol extract =111.62 ± 0.01 mg GAE/g; methanol extract =139.99 ± 0.01 mg GAE/g; water extract =105.66 ± 0.01 mg GAE/g).

**Table 5:** **Correlation between antioxidant activity of different extracts of selected Sudanese plants and the total content of flavonoids and phenolics in the extracts**

Plants	Part used	Solvent used	DPPH (IC _50_ in μg/ml)	Total flavonoids (mg/g)	Total phenolics (mg/g)
*Indgosfera spinosa*	Stem	n-Hexane	>1000	28.1 ± 0.00	ND
		Ethanol	192.63	40.2 ± 0.04	115.4 ± 0.01
		Methanol	140	36.6 ± 0.01	161.4 ± 0.03
		Water	>1000	13.3 ± 0.01	78.5 ± 0.001
*Nicotiana gluaca*	Leaves	n-Hexane	>1000	88.1 ± 0.01	ND
		Ethanol	54.76	77.1 ± 0.00	111.6 ± 0.01
		Methanol	13.437	64.5 ± 0.002	139.9 ± 0.01
		Water	30.05	57.6 ± 0.001	105.6 ± 0.01
	Stem	n-Hexane	>1000	58.6 ± 0.02	3.46 ± 0.01
		Ethanol	160.94	13.1 ± 0.01	ND
		Methanol	119.45	17.9 ± 0.01	41.02 ± 0.003
		Water	591.98	ND	12.5 ± 0.02
*Tephrosia apollinea*	Aerial part	n-Hexane	>1000	66.9 ± 0.01	ND
		Ethanol	120.22	78.1 ± 0.00	ND
		Methanol	48.803	10.7 ± 0.01	25.7 ± 0.00
		Water	2835.07	23.5 ± 0.02	23.5 ± 0.01
*Tamarix nilotica*	Leaves	n-Hexane	>1000	67.8 ± 0.00	ND
		Ethanol	880.70	13.1 ± 0.04	15.3 ± 0.04
		Methanol	12.62	24.9 ± 0.02	333.9 ± 0.01
		Water	67.53	22.6 ± 0.001	171.6 ± 0.02
*Combretum hartmannianum*	Leaves	n-Hexane	>1000	42. ±0.002	ND
		Ethanol	146.35	45.2 ± 0.001	169.2 ± 0.02
		Methanol	14.067	57.7 ± 0.001	392.8 ± 0.01
		Water	967.55	27.6 ± 0.02	268.2 ± 0.03
	Bark	n-Hexane	>1000	42.1 ± 0.01	1.9 ± 0.01
		Ethanol	9.47	28.1 ± 0.02	404.1 ± 0.06
		Methanol	28.91	24.2 ± 0.02	96.5 ± 0.05
		Water	22.47	29.7 ± 0.01	523.3 ± 0.01
*Capparis decidua*	Stem	n-Hexane	212.56	67.5 ± 0.02	85.2 ± 0.01
		Ethanol	900.58	8.5 ± 0.02	19.9 ± 0.01
		Methanol	76.38	4.6 ± 0.02	40.3 ± 0.2
		Water	>1000	ND	335.7 ± 0.01

ND = Not Detected. The results are presented as mean ± SEM. Each experiment was repeated three times; (n =3).

### Total flavonoids contents in the extracts

The total flavonoids contents in each extract were determined from linear regression equation of calibration curve obtained from the different concentrations of quercetin [y = 0.0032 + 0.0669 (R^2^ = 0.986)] and the results were expressed as mg quercetin equivalent/g of extracts (Table 5). Hexane extracts of all the tested plants and their parts showed higher content of flavonoids than compared to the other solvent extracts. However, on average all the extracts demonstrated considerably high amount of flavonoids in *N. glauca* leaves with hexane extract =88.150 ± 0.01 mg quercetin equivalent/g, ethanol extract =77.083 ± 0.00 mg quercetin equivalent/g, methanol extract =64.583 ± mg quercetin equivalent/g and water extract =57.667 ± mg quercetin equivalent/g. Extracts of aerial parts of *T. apollinea* also showed significant level of flavonoids with hexane extract =66.950 ± 0.01 mg quercetin equivalent/g, ethanol extract =78.092 ± 0.00 mg quercetin equivalent/g, methanol extract =10.708 ± 0.01 mg quercetin equivalent/g and water extract =23.575 ± 0.02 mg quercetin equivalent/g. The total flavonoid contents measured in the extracts is given in the Table 5.

### DPPH scavenging effect of the extracts

IC_50_ values of DPPH scavenging activity of the extracts is tabulated in Table 5. On average, the extracts prepared from methanol demonstrated the most potent antioxidant activity, whereas the extracts prepared from the solvent hexane displayed poor DPPH scavenging activity as the IC_50_ values estimated to be more than 1000 μg/ml. Among the tested plants, *C. hartmannianum* stem bark exhibited significant (p < 0.01) antioxidant activity as lowest IC_50_ values were calculated for ethanol extract (IC_50_ = 9.47 μg/ml), methanol extract (IC_50_ = 28.91 μg/ml) and water extract (IC_50_ = 22.47 μg/ml). Similarly, *N. glauca* leaves also demonstrated significant (p < 0.05) antioxidant activity. The IC_50_ values for ethanol, methanol and water extracts of *N. glauca* leaves were calculated as 54.76, 13.437 and 30.05 μg/ml, respectively. The other plants displayed either moderate or insignificant DPPH scavenging activity (Table 5).

## Discussion

Increasing evidences in both experimental and clinical studies suggest that angiogenesis and oxidative stress play a cumulative role in the pathogenesis of malignancy. Angiogenesis, a process of formation of new blood vessel from pre-existing vessels, strongly implicates in the metastatic carcinogenesis. The role of reactive oxygen species (ROS) in tumorous angiogenesis has been extensively investigated and various connections have been established [25]. ROS can function as signaling molecules to mediate various angiogenic-related responses such as cell proliferation, differentiations and migration [26, 27].

Natural anticancer medicines discovered from various medicinal systems that have been derived from traditional knowledge and practiced in many countries. Similarly, many herbal extracts and products are traditionally being used in Sudan for the treatment of cancer [28]. However, such medicinal plants have not gained clinical importance as botanicals due to the lack of systematic and scientific evidence presented through the suitable standard experimental procedures. In the present study six indigenous anticancer medicinal plants (*I. spinosa, N. glauca, T. apollinea, T. nilotica, C. hartmannianum* and *C. decidua*) from Sudan were studied. Since the people of Sudan have long been using these plants as either food or medicine, the plants considered as an integral part of the local pharmacopoeia. In the present study, different parts of the plants were selected to prepare 32 extracts using sequential extraction method with 4 solvents of different polarity. The rationale for performing extractions from non-polar to polar solvents is to confirm and validate the bio-efficacy in the aqueous extractions performed in the traditional manner in the form of tonics and aqueous pastes. In addition, the extracts with different polarities provide an idea about the specific phytochemical groups of the active principles present in the extracts.

This study aimed to evaluate the anti-carcinogenic activities of the 6 Sudanese medicinal plants and to correlate these activities with phytochemical analysis and antioxidant capability. Two assays were used to assess the anti-carcinogenic properties of the plant extracts. One is the MTT assay which provides a simple method for determination of cell’s viability via mitochondrial activity in living cells and other one is the rat aorta ring assay, which is based on the ability of the aortic wall to produce neo-vessels in bio-matrix gels after mechanical injury or angiogenic factor stimulation.

The results of rat aortic ring assay showed that, out of 6 tested plants, *T. apollinea*, *C. hartmannianum* and *T. nilotica* showed strong inhibitory effect (more than 60%). Particularly, the ethanol extract of aerial parts of *T. apollinea* demonstrated highest anti-angiogenic activity by inhibiting 100% of the aortic microvessels. Noteworthily, all the extracts of *C. hartmannianum* displayed significant anti-angiogenic activity.

Whereas, results of the cytotoxic assay showed that among all the extracts, the highly non-polar solvent extract i.e., hexane extract demonstrated higher cytotoxic activity than the other solvent extracts. Among the 6 plants, the hexane extracts of *N. glauca* leaves and stem bark exhibited most potent anti-proliferative effect on all the tested cancer cell lines. However, for the plant *T. apollinea*, both non-polar (hexane) and polar (ethanol) extracts displayed selective cytotoxicity towards hormone dependent breast cancer cell line (MCF-7). Consistently, again the extracts of *C. hartmannianum* leaves and stem bark exhibited strong inhibitory effect against the proliferation of HCT-116 and MCF-7 cells. Noteworthily, the extracts of the most effective plants (*N. glauca*, *T. apollinea* and *C. hartmannianum*) did not produce significant cytotoxic effects against the normal cell lines (HUVEC and CCD-18Co).

Altogether, the most biologically active plants which showed significant antiangiogenic as well as cytotoxic activities were *N. glauca*, *T. apollinea*, *C. hartmannianum* and *T. nilotica*. These findings were further supported by the results of DPPH scavenging activity. The results showed that, most of the extracts of *C. hartmannianum* displayed strong DPPH quenching ability with lowest IC_50_ (9 μg/ml), which was followed by *N. glauca*, *T. nilotica* and *T. apollinea* (Table 5).

The antiangiogenic and anti-proliferative effects of the plants may be due to their potential antioxidant activity, which further attributes to the collective contribution of phenolics and flavonoids present it the respective extracts. The findings of the present study revealed that these plants are enriched with phenolic and flavonoid contents than compared to the other plants which have shown less bio-efficacy. On average, the plants *N. glauca*, *T. apollinea*, *C. hartmannianum* and *T. nilotica* exhibited high antioxidant activity in DPPH free radical scavenging assay. This may support the traditional usage of these plants to improve complications such oxidative stress in cancer and other diseases. Many health beneficial effects of flavonoids are attributed to their ability to act as antioxidants. Research studies have shown that flavonoids as single electron donors can stabilize and scavenge the free radicals, which in conditions of oxidative stress may initiate angiogenesis or carcinogenesis. Similarly, phenolics have strong capability to interfere in a series of physiological events in biological systems, including those relating to oxidation processes [29].

Phenolics and flavonoids mostly found in plants are reported to have numerous biological effects including antioxidant, anti-neovascularization, antiproliferation and anticarcinogenic properties and are therefore considered for their important dietary roles as antioxidants and chemoprotective agents. Recently, intensive research has been focused on studying the naturally occurring phenolics and flavonoids that are able to decrease the generation of reactive oxygen species (ROS) in biological system. Oxidative stress contributed by ROS plays a critical role in the pathologies related with chronic disease such as cancer and excessive vascularization [30]. ROS-induced development of cancer involves malignant transformation due to DNA mutations and altered gene expression through epigenetic mechanisms which in turn leads to the uncontrolled proliferation of cancerous cells. Further, high levels of ROS are observed in various cancerous cells and a number of accumulating evidences [31–33] suggest that ROS function as key signaling molecules to stimulate various growth-related responses that eventually initiate angiogenesis and tumorigenesis [27]. Several studies demonstrated a significant role of phenolics in growth inhibition of breast, colon, prostate, ovary, endometrium and lung cancer cells [34, 35].

The present study confirmed that the extracts of *N. glauca*, *T. apollinea* and *C. hartmannianum* demonstrated selective cytotoxicity towards human breast and colon cancer cell lines while being less cytotoxic against the normal cells. Such selective cytotoxic activity suggested that the active substances interact with special cancer-associated receptors or cancer cell special molecule, thus triggering some mechanisms that cause cancer cell death [36]. In observation under EVOS f1 digital microscope, typical apoptotic characteristics were observed, including cell membrane blebbing, loss of pseudopodia-like cellular projections, nuclear condensation, and separated apoptotic bodies (Figure 3). In addition, the treated MCF-7 cells displayed typical signs of apoptosis such as shrinkage of cells, chromatin condensation and crescent shaped nuclei (Figure 3, MCF-7 row). Several reports have revealed that flavonoids are able to inhibit the growth of cancer cells *in vitro* [37–39]. These findings are confirmed by several *in vivo* studies [40]. Flavonoids exert their anticancer action through affecting key mechanisms involved in cancer pathogenesis. Flavonoids are effective antioxidant and antiangiogenic agents. In initial stages, they inhibit metabolic activation of carcinogens. In progression phases they induce apoptosis, inhibit angiogenesis, cancer cell proliferation and tumor metastasis [41].

The magnitude of phenolic compounds in redox system acts either as reducing agents, hydrogen donators, singlet oxygen quenchers or in some cases as a metal chelating agents [42] thereby, the free radicals which generated in the metabolic pathways could be neutralized by phenolic compounds [43]. From the results obtained in this study, there is an obvious correlation between antiangiogenic and antioxidant activity, since the polyphenols inhibit the initiation and progression of angiogenesis [44–46]. Therefore, plant polyphenols may play an important role in halting angiogenesis, as well as have ability to prevent oxidant potentials of free radicals as natural source of antioxidants.

Results of the present study were completely in agreement with the study reported by Mariod et al., [47] on the various extracts of *C. hartmannianum* for its antioxidant property and total phenolic content. In the present study, *C. hartmannianum* demonstrated strong antiproliferative and antiangiogenic activities. It is reported that *C. hartmannianum* has strong capability to inhibit tyrosine kinase [48]. Tyrosine kinase is an important cellular signaling protein which has essential and critical role in several biological activities including cell proliferation and angiogenesis [49]. Several tyrosine kinase inhibitors such as, bevacizumab, sunitinib, sorafenib and pazopanib were recently approved for treatment of patients with malignant carcinoma [50, 51]. In addition, many other anti-angiogenic tyrosine kinase inhibitors are being studied in phase I-III clinical trials in order to validate their beneficial anti-tumor, anti-metastatic and anti-angiogenic activities in human beings [52].

In this study, another herb that has shown a promising cytotoxicity on the tested cancer cell lines was *N. glauca*. Ibrahim and El-Sharkawy [53] reported the antioxidant activity and presence of phenolics and flavonoids in the leaves of *N. glauca* however, the present study reported that even stem bark extracts of the plant has remarkable antioxidant activity which could be attributed to the presence of high levels of phenolics and flavonoids in it.

The present study showed that both non-polar and polar extracts of aerial part of *T. apollinea* (Figure 3) exhibited selective antiproliferative effect against breast cancer cell line MCF-7. However, in our previous study [54] we reported the isolation of (-)-pseudosemiglabrin from aerial parts of *T. apollinea* and its antiproliferative activity on prostate, leukemia, breast and colon cancer cells. In the present study, the anticancer and antiangiogenic activities of nonpolar (hexane) extracts could be partly attributed to the high levels of flavonoid. It is well known that the plant-flavonoids demonstrated promising anticancer and antiangiogenic activities [55]. Several studies [56] reported the presence of complex prenylated flavones and phenolic compounds in *T. apollinea* and thus the antiangiogenic and antiproliferative properties of the herb recorded in the present study can be attributed to the cumulative effects of such bioactive constituents.

For the first time the present study reported the cytotoxic potentials of leaves of *T. nilotica* against human colon (HCT-116) and breast (MCF-7) cancer cells. However, the earlier reports mentioned that *T. nilotica* has selective cytotoxic potential against liver cell carcinoma (Huh-7), while being non-toxic to other cancer cells [57]. Nevertheless, the results of the present study agree in part with the previous findings [57] in the sense that *T. nilotica* has noticeable DPPH quenching capability. These findings are in agreement with some earlier reports [58, 59] wherein, various types of phenolic constituents such as phenolic glyceride, phenolic lactone, phenolic aldehydes and dimeric phenols were isolated from the of *T. nilotica*.

Anti-angiogenic agent could target the cancer or endothelial cells at any of the steps necessary for carcinogenesis or neovascularization, such as proliferation, differentiation, migration, or tube formation [60]. Angiogenesis inhibitors act by actively promoting apoptosis in cells. *In vitro* and *in vivo* investigations have proved that many endogenous antiangiogenic compounds induce cytotoxicity via apoptotic cellular death [61].

The results of the present study could be very helpful as preliminary data in the search for new antitumor compounds from the tested Sudanese traditional medicinal plants. These plants have the potential to be chemically standardized and used as herbal medicines or developed into pharmaceutical drugs for the treatment of angiogenesis-dependent human ailments such as cancer and other hyperproliferative disorders. Bioassay-guided phytochemical and pharmacological studies are under investigation in an attempt to isolate and characterize the active constituents from the extracts of the plants which have shown promising antiangiogenic and anticancer properties.

## Conclusion

In conclusion the present study revealed that among the six Sudanese medicinal plants tested, *N. glauca*, *T. apollinea*, *C. hartmannianum* and *T. nilotica* found to be most biologically effective herbs with significant antiangiogenic and antineoplastic effects. The biological activities observed in the study could be attributed to the antioxidant and higher phenolic and flavonoid contents of the plants. Furthermore, the extracts of these plants displayed either negligible or insignificant cytotoxicity against the tested human normal cells. Thus, these herbs could be considered as promising candidates for the development of novel chemopreventive or chemotherapeutic formulations with reduced side effects. The results obtained in the present study justify the traditional use of these medicinal plants to cure neoplasia. However, further studies to isolate the active compounds and to investigate the mode of action using *in vivo* xenograft experimental models are warranted against pathological neovascularization and tumor malignancy.
